# Targeted Detection of G-Quadruplexes in Cellular RNAs**

**DOI:** 10.1002/anie.201500891

**Published:** 2015-04-23

**Authors:** Chun Kit Kwok, Shankar Balasubramanian

**Affiliations:** The University of Cambridge, Department of Chemistry, Lensfield RoadCambridge, CB2 1EW (UK)

**Keywords:** cations, G-quadruplexes, ligand effects, reverse transcription, RNA structures, telomerase RNA

## Abstract

The G-quadruplex (G4) is a non-canonical nucleic acid structure which regulates important cellular processes. RNA G4s have recently been shown to exist in human cells and be biologically significant. Described herein is a new approach to detect and map RNA G4s in cellular transcripts. This method exploits the specific control of RNA G4–cation and RNA G4–ligand interactions during reverse transcription, by using a selective reverse transcriptase to monitor RNA G4-mediated reverse transcriptase stalling (RTS) events. Importantly, a ligation-amplification strategy is coupled with RTS, and enables detection and mapping of G4s in important, low-abundance cellular RNAs. Strong evidence is provided for G4 formation in full-length cellular human telomerase RNA, offering important insights into its cellular function.

G-quadruplex (G4) nucleic-acid structures play pivotal roles in the regulation of a myriad of cellular processes,[1] and have been demonstrated to be versatile scaffolds for ligand and biosensor development.[2] Recently, G4 s were visualized using G4-specific antibodies in human cells and tissues, and G4 ligands can stabilize such structures in cells.[3] Approaches for RNA G4 identification in cellular RNA have been largely limited to computational predictions, using algorithms such as Quadparser and QGRS.[4] Recent discoveries have started to specifically reveal important roles for RNA G4s in cells.[5] It is now essential to establish ways to explicitly map RNA G4 formation and location in cellular transcripts to establish their role(s) and facilitate small-molecule intervention strategies.

G4s form from guanine-rich sequences which self-assemble by stacked G-tetrads that are further stabilized by cations such as K^+^ (Figures 1 and 2 A).[6] G4 structures exhibit characteristic features in circular dichroism, UV-thermal melting analysis, fluorescence, and NMR spectroscopy.[7] The synthetic or in vitro transcribed (IVT) RNA can be subjected to in-line probing to detect the presence of RNA G4s.[8] Compared to the abundant rRNAs and tRNAs, most cellular transcripts are of lower abundance, therefore it is challenging to adapt existing approaches to probe the formation and location of G4s in full-length cellular RNAs.

**Figure 1:**
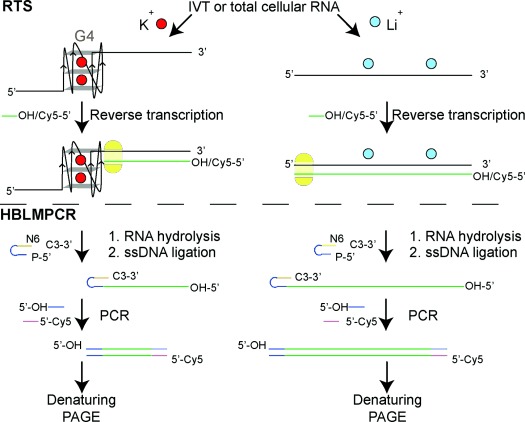
Targeted detection of G4s using RTS or RTS-HBLMPCR. IVT or total cellular RNA was used. Reverse transcription was conducted with Cy5-labeled/unlabeled gene-specific primer (green) for RTS/RTS-HBLMPCR, respectively. For ligand treatment experiments, ligand was added prior to reverse transcription (not explicitly illustrated). After reverse transcription, RNA was hydrolyzed, and the Cy5-labeled cDNAs were analyzed by denaturing PAGE. For RTS-HBLMPCR, the unlabeled cDNAs were ligated to a single-stranded DNA (ssDNA) linker (blue and orange). The ligated cDNA was PCR with linker-specific unlabeled forward primer (blue) and gene-specific Cy5-labeled reverse primer (pink). The PCR products were analyzed by denaturing PAGE.

**Figure 2:**
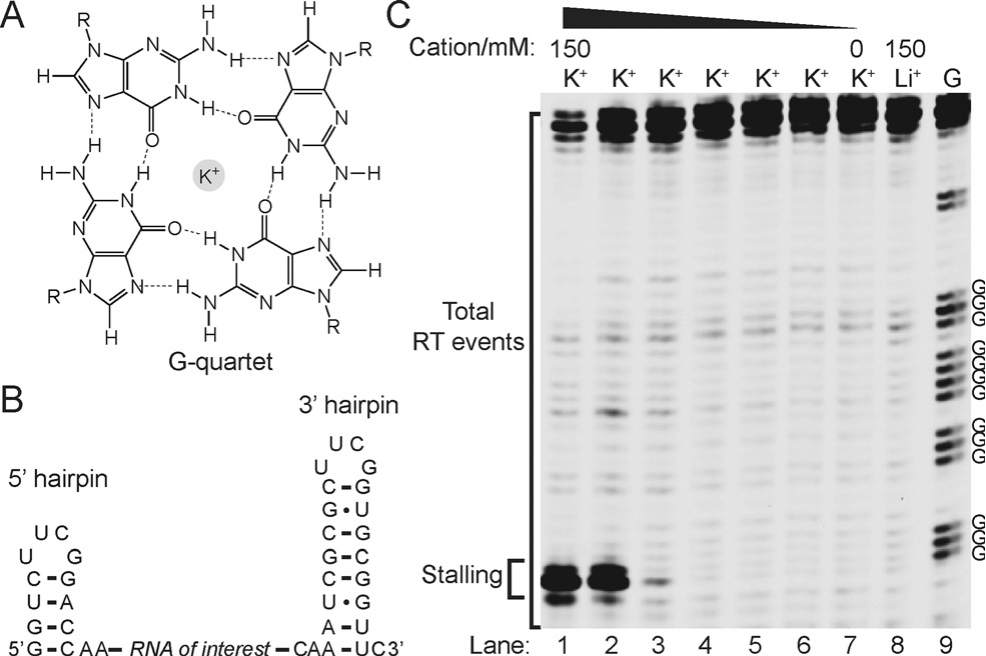
RNA G4-mediated RTS is controlled by cations and [K^+^]. A) Chemical structure of G-quartet. The K^+^ sits in the core of the G-quartet. B) IVT RNA design used. The RNA of interest is designed within a 5′ hairpin and 3′ hairpin. C) The example shown here is a RNA G4 found in the 5′ UTR of human *NRAS*. The labeled Gs are the four G-tracts from the *NRAS* G4. In lanes 1 and 8, 150 mM K^+^ and 150 mM Li^+^, respectively, was used for the reverse transcription (see materials and methods). Lanes 1–6 show the reverse transcription result over a 10-fold dilution series of [K^+^], that is, lane 1 is 150 mM K^+^, lane 2 is 15 mM K^+^, and so on. Lane 7 has no added K^+^. Lane 9 is a G-ladder by dideoxy C sequencing. The stalling and total RT events were bracketed and used for RTS calculations.

Herein, we show that RNA G4-mediated reverse transcriptase stalling (RTS) can be rationally controlled in a cation- and G4 ligand-dependent fashion (Figure 1). Notably, we have integrated RTS with hybridization-based ligation-mediated PCR (RTS-HBLMPCR) to demonstrate and positionally map RNA G4 formation in cellular transcripts. This new approach has the sensitivity to enable G4 mapping of functionally important transcripts at natural abundance levels (Figure 1).

To identify a suitable reverse transcriptase and conditions for RNA G4 detection and mapping, we rationally designed the IVT RNA construct such that the RNA of interest is within a structure cassette, which contains a 5′ hairpin that serves as internal RTS control, and a 3′ hairpin for primer binding (Figure 2 B).[9] Using a well-studied RNA G4 which is present in the 5′UTR of human *NRAS*,[10] **GGG**A**GGGG**C**GGG**UCU**GGG**, (see Table S1 in the Supporting Information), we evaluated a series of commercially available reverse transcriptases such as SuperScript III (Life Technologies) and AMV (Roche) using the reverse transcription buffer provided (containing K^+^) and observed strong RTS near the RNA G4 (see Figure S1 in the Supporting Information). This observation is similar to a previous report,[11] thus confirming that in the presence of K^+^, reverse transcriptases stall at G4 sites frequently. A closer inspection of the stalling position revealed that it usually occurs one nucleotide (nt) before the 5′ end of the G4 of the reverse transcribed cDNA, and corresponds to one nt after the 3′ end of G4 in RNA (Figures 1 and 2 C). Next, we sought conditions to alleviate RTS at G4s. Given that G4 stability has a strong cation dependence,[12] in the order K^+^>Na^+^>Li^+^, we performed ion-dependent reverse transcription and showed that one of the enzymes, Superscript III reverse transcriptase, stalled at the G4 site in a 150 mM K^+^-containing buffer, but not in either a 150 mM Na^+^- or Li^+^-containing buffer (same ionic strength; Figure S1). Specifically, 16-fold higher stalling was observed in the presence of K^+^ versus Li^+^ (Figure 2 C; see Table S2 in the Supporting Information), where we define the RTS effect as the fraction of stalling observed as a proportion of the total RT events under the conditions employed (e.g. K^+^ in this case) over Li^+^ condition (Figure 2 C, lanes 1 and 8). Moreover, we demonstrated that the RTS effect in *NRAS* G4 is progressively suppressed with decreasing [K^+^] (Figure 2 C, lanes 1-7), thus corroborating that RTS correlates to G4 stability. Further experiments confirmed the RTS effect with other well-known and validated RNA G4s from human genes including *TRF2*,[13] *MT3*,[14] *BCL2*,[15] and others (see Figures S2–S8 in the Supporting Information),[16] whereas control RNA structural motifs, such as the hairpin (HP) and pseudoknot (PK), did not display RTS effect (see Figures S9–11 in the Supporting Information). This data strongly indicates that the observed RTS is G4-specific. These results show that ion-dependent RTS can detect and map RNA G4s in the context of extended transcripts.

The targeting of G4 structures using small molecules offers the potential to intervene with biological processes.[3a,b,^17^] We are therefore interested to see whether RTS could probe RNA G4 targeting by a stabilizing ligand, as this could validate the molecular target and provide insights into ligand selectivity. We started by using a strong G4 stabilizing ligand called pyridostatin (PDS; Figure 3 A)_._[17] Using the *NRAS* G4 RNA system under Li^+^ conditions, which do not promote G4 formation, we observed a PDS concentration-dependent increase in RTS (Figure 3 B, lanes 2–7), thus yielding a five-fold RTS effect at 1 μM PDS (see Table S2). In a control experiment we introduced excess of a DNA G4 competitor (30 μM c-MYC) for PDS binding and observed a reduction in RTS (Figure 3 B, lane 8), thus verifying that the ligand effect was a result of G4 recognition and stabilization. We also demonstrated that the excess DNA G4 spiked in did not affect the reverse transcription (Figure 3 B, lane 9). To provide insights into the effect of different G4 ligands on RNA G4, we tested other G4 ligands such as cPDS,[2a PhenDC3,[18] and TMPyP4[19] under the same dosage at 1 μM (see Figure S12 in the Supporting Information). We noted that higher G4 ligand concentration started to inhibit the activity of reverse transcriptase (data not shown). We found that cPDS (see Figure S12A) has a similar structure to that of PDS and showed almost the same RTS effect, whereas PhenDC3 and TMPyP4 (see Figure S12A), which have quite different structures from PDS, exhibited either weaker or no RTS effect, respectively (see Figure S12B). We also performed the same experiments using telomeric *TERRA* RNA G4,[20] and observed an identical trend for the RTS effect, that is, PDS≈cPDS>PhenDC3>TMPyP4 (see Figure S12C). These findings suggest that these G4 ligands each have a different potential for targeting RNA G4s in extended transcripts.

**Figure 3:**
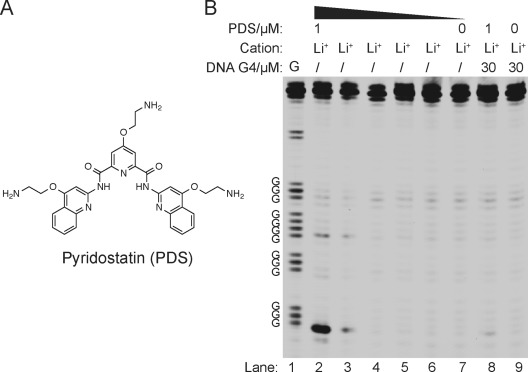
RNA G4-mediated RTS can be controlled by pyridostatin. A) Chemical structure of PDS. B) The [PDS]-dependence experiments on *NRAS* G4 under Li^+^ conditions. Lanes 2–6 show the reverse transcription result over a five-fold dilution series of [PDS] under 150 mM Li^+^ conditions, that is, lane 2 is 1 μM PDS, lane 3 is 0.2 μM PDS, and so on. Lane 7 has no added PDS. Lane 8 is conducted the same way as lane 2, except that 30 μM of DNA G4 is added to compete for PDS and alleviate the RTS. Lane 9 is conducted the same way as lane 7, except that 30 μM of DNA G4 is added. Lane 1 is G ladder by dideoxy C sequencing.

Taking the results of RNA G4-cations and RNA G4-PDS together, we observed a 16- (K^+^/Li^+^), 19- (K^+^[PDS]/Li^+^), and 5-fold (Li^+^[PDS]/Li^+^) RTS effect for *NRAS* G4 (Figure 4 A and see Table S2). Similar cation- and PDS-dependent RTS trends were found for other RNA G4s, but were absent for the RNA hairpin and RNA pseudoknot constructs (see Figures S2–11 and Table S2), thus supporting that we are observing a G4 structure-specific RTS effect (Figure 4 B). The results of all RNA constructs are summarized in Table S2.

**Figure 4:**
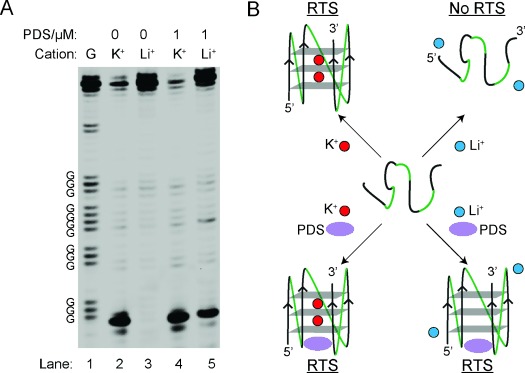
Overall results and proposed model for RNA G4-mediated RTS. A) Lanes 2 and 3 show the reverse transcription result under 150 mM K^+^ or Li^+^. Lanes 4 and 5 show the reverse transcription result under 150 mM K^+^ or Li^+^ with 1 μM PDS. Lane 1 is a G ladder generated by dideoxy C sequencing. B) Proposed model for G4-mediated RTS. G4 can be stabilized by K^+^ and/or PDS, and induces RTS.

Whilst the above results were performed in IVT RNAs, detecting and mapping RNA G4 within a specific transcript in total cellular RNA is more challenging. For low-abundance cellular transcripts, the cDNAs generated by reverse transcription are scarce and cannot be detected by RTS assay without further amplification. To achieve this, we coupled the RTS assay with ligation-mediated PCR[21] (LMPCR), in which single-stranded DNA (ssDNA) ligation was first performed to ligate the cDNAs to a known DNA linker, and then followed by PCR (Figure 1). For the ssDNA ligation step, we implemented a hybridization-based (HB) ligation strategy[21b and designed a ssDNA linker containing a stable hairpin with a degenerate hexamer (N6) tail for efficient hybridization and ligation to incoming cDNAs (Figure 1, ssDNA ligation step; and see Figure S13 in the Supporting Information). Our ligation result showed that the ligation yield was up to about 90 % in 2 hours (see Figure S13), and shows that HB ligation is robust and nearly quantitative.

We then applied RTS-HBLMPCR to investigate RNA G4 formation in human telomerase RNA (*hTERC*), a 451 nt long noncoding RNA (lncRNA) critical for the regulation of telomere length.[22] *hTERC* RNA is present at only about 1000 copies/cell in most human cells,[23] thus making it a challenging test case for our method. The low-abundance of *hTERC* relative to *5.8S* rRNA (ca. 200 fold higher) and actin *ACTB* mRNA (ca. 10-fold higher) in *HeLa* cells was evident by qRT-PCR (see Figure S14 in the Supporting Information). Cation- and PDS-dependent RTS-HBLMPCR were conducted (Figure 5 A), and revealed a number of key findings. First, a strong RTS effect near the 5’ end of *hTERC* RNA was detected, in which a close examination on the sequence alone suggested putative G4 formation (Figure 5). Thus, our RTS-HBLMPCR result here provides robust and direct experimental evidence for the formation of G4s in full-length *hTERC* in total cellular RNA. Second, two major stalling bands were observed (Figure 5, asterisks), thus indicating that more than one RNA G4 was formed, which can be reasoned by the fact that it has nine G-tracts available for involvement in G4 formation (Figure 5 B, bolded). Based on the stalling location, it is most likely that two major RNA G4s (nt 1–17 and nt 21–34) are formed in full-length *hTERC*. Recently, the nt 1–17 RNA G4 has been solved by NMR spectroscopy,[24] which supported our finding here. These results also suggest RTS and RTS-HBLMPCR can detect multiple or tandem RNA G4 s in a single experiment, and could be useful given the prevalence of RNA G4s in human transcriptome.[4b Lastly, the result of full-length cellular *hTERC* (Figure 5) was comparable to *hTERC* IVT RNA (see Figure S8), thus showing that the *hTERC* RNA G4s are stable and the flanking sequences did not preclude G4 formation.

**Figure 5:**
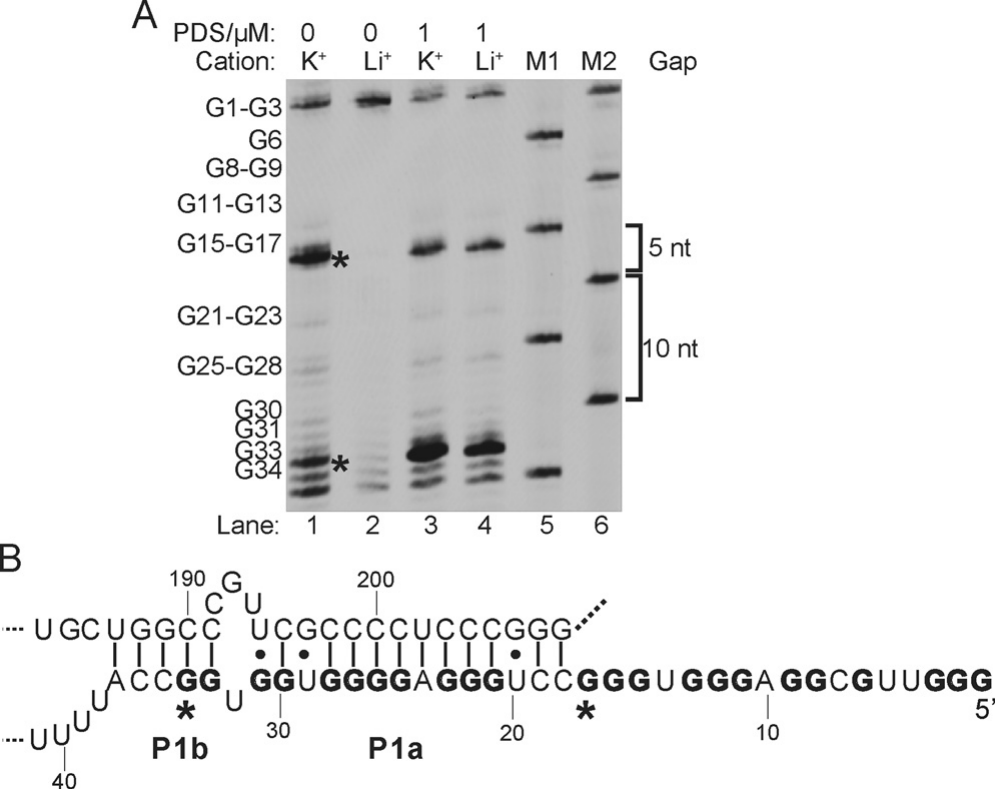
RTS-HBLMPCR reveals G4 formation in *hTERC*. A) Cation- (Lanes 1 and 2) and PDS-dependent (Lanes 3 and 4) RTS-HBLMPCR on *hTERC*. Lanes 5 and 6 are size markers. B) Phylogenetic structure of *hTERC*. The two major stalling points are shown in asterisks. The G-tracts involved in G4s are in bold.

The phylogenetic RNA secondary structure of *hTERC* shows that some of the G tracts are involved in the formation of a P1 helix (Figure 5 B), a key element defining the template boundary of reverse transcription in telomerase.[25] UV-melting experiments on the *hTERC* G4 sequence (nt 1–41) gave a melting temperature (Tm) of greater than 80 °C at 100 mM K^+^.[26] The addition of the complementary sequence (nt 184–208) yielded a Tm of 73 °C for the duplex.[26] Combined with our RTS-HBLMPCR result here (Figure 5 A), it is likely that the G4s and P1 helix coexists in full length *hTERC*, with G4s being more stable than the P1 helix (weak GU pairs and internal loop) under physiological conditions which have high [K^+^], about 150 mM. We cannot rule out the possibility that both structures could form simultaneously with a quadruplex comprising a C bulge (nt 1–17).[24] Recent protein binding and RNA mutational studies have suggested that the RNA G4-specific helicase DHX36 is involved in the binding and unwinding of a putative RNA G4 located near the 5’ end of *hTERC*,[27] and that the G4 may facilitate the accumulation of mature *hTERC* needed for telomere maintenance.[27a Our finding here that G4s are stable and detectable in full-length cellular *hTERC* support the fact that enzymes which can resolve G4 in RNA, such as DHX36,[27] may play a role in ensuring active *hTERC* RNA conformation for telomerase function.

In summary, we introduce the RTS-HBLMPCR approach for probing G4 formation and location in full-length low-abundance cellular RNAs. We exemplify the approach by detecting and mapping G4 formation in the biologically important cellular lncRNA, *hTERC*. Moreover, we show that the approach is applicable to detect and validate RNA targets for G4 ligands. In the future, we will adapt the approach described here to map G4s throughout the transcriptome.
