# Supplementary material for: Targeted Detection of G-Quadruplexes in Cellular RNAs
Source: Angew Chem Int Ed Engl. 2015 Apr 23;54(23):6751–4. doi: 10.1002/anie.201500891 (PMC4510783; doi:10.1002/anie.201500891)
Supplement: Supplementary file 1 [file anie0054-6751-sd1.pdf]

Angewandte Chemie
Eine Zeitschrift der Gesellschaft Deutscher Chemiker

Supporting Information

German Edition: DOI:

Targeted Detection of G-Quadruplexes in Cellular RNAs**

*Chun Kit Kwok and Shankar Balasubramanian**

anie_201500891_sm_miscellaneous_information.pdf

Supporting Information

Materials and methods

Table S1. DNA and RNA sequences used in this study.

Table S2. RTS effect of all IVT RNA constructs.

Figure S1. Identification of selective reverse transcriptase and reverse transcription conditions for RNA G4-mediated RTS.

Figure S2. Cation- and PDS-dependent RTS on *TRF2* IVT RNA.

Figure S3. Cation- and PDS-dependent RTS on *MT3* IVT RNA.

Figure S4. Cation- and PDS-dependent RTS on *BCL2* IVT RNA.

Figure S5. Cation- and PDS-dependent RTS on *ADAM10* IVT RNA.

Figure S6. Cation- and PDS-dependent RTS on *ZIC1* IVT RNA.

Figure S7. Cation- and PDS-dependent RTS on *TERRA* IVT RNA.

Figure S8. Cation- and PDS-dependent RTS on *TERC* IVT RNA.

Figure S9. Cation- and PDS-dependent RTS on HP IVT RNA.

Figure S10. Cation- and PDS-dependent RTS on PK IVT RNA.

Figure S11. Cation- and PDS-dependent RTS on *TAR* HP IVT RNA.

Figure S12. G4 ligand-dependent RTS on *NRAS* and *TERRA* IVT RNAs.

Figure S13. Single-stranded DNA (ssDNA) ligation construct and efficiency test using human *5.8S* rRNA.

Figure S14. qRT-PCR result on human *5.8S* rRNA, actin *ACTB* mRNA and *TERC* lncRNA

References.

Materials and methods

Preparation of DNA oligonucleotides

All DNA oligonucleotides were purchased from Integrated DNA Technologies (IDT). The fluorescently-labelled DNAs were HPLC purified. The quality of the each oligonucleotide was checked by ESI-MS by IDT, with a single peak at the expected size, so all DNA oligonucleotides were used without further purification.

Preparation of in vitro transcribed RNAs

In vitro transcribed (IVT) RNAs were made using pre-annealed DNA hemiduplex and MEGA shortscript T7 Kit (Ambion) following the manufacturer's protocol. Each RNA was purified using the 7 M urea, 15 % denaturing acrylamide gel (Life Technologies) and the desired RNA gel band was sliced under brief UV shadowing. The gel piece was crushed and soaked in 1X 10 mM Tris pH 7.5, 1 mM EDTA, 800 mM LiCl (1X TEL_{800}) as described previously[1]. The use of 800 mM LiCl over the commonly used 250 mM NaCl was to minimize the Na^+-induced G-quadruplex formation before the experiment. The mixture was under constant rotary shaking overnight at 4 °C. Next, the mixture was filtered against 0.2 μM filter with a syringe, and 3X volume of 100% ethanol and 1 μL of 15 mg/ml glycol blue (Life Technologies) was added and mix thoroughly before putting into dry ice for ethanol precipitation. After an hour, the slurry was centrifuged at 15,000 rpm for 15 min, and then washed one time with 70 % ethanol to remove residual salt. The RNA was dried down in a speedvac at room temperature for 10 min. The dried RNA was resuspensed in nuclease-free water and quantified by UV spectroscopy. The IVT RNA was then stored at -20 °C before use.

Preparation of total cellular RNA

Human *HeLa* cells were grown in with DMEM growth media (Sigma) supplemented with 10 % fetal bovine serum (Sigma). Cells were washed with PBS, detached from cell surface by trypsin-EDTA (Invitrogen). Trypsin was inactivated by adding 2X volume of above-mentioned media. The cells were spun down at 1300 rpm for 3 min. Total cellular RNA from cell pellet was extracted using Qiagen RNeasy Plus Mini Kit following manufacturer's protocol. The total cellular RNA was stored at -20 °C.

Reverse transcription

Three to five pmol of IVT RNA or 1 μg of total cellular RNA was added up to 4.5 μL with nuclease-free water, and 1 μL of 5 μM Cy5 fluorescently-labelled (for RTS) or 1 μL of 2.5 μM unlabelled (for RTS-HBLMPCR) gene-specific DNA primer was subsequently added. The mixture was heated at 75 °C for 3 min, followed by 35 °C for 5 min. At the beginning of the 35 °C step, 3 μl of reverse transcription buffer was added to give a final concentration of 150 mM KCl, 4 mM $MgCl_2$, 20 mM Tris pH 7.5, 1 mM DTT, and 0.5 mM dNTPs. For cation-dependent experiments, either 150 mM KCl or NaCl or LiCl was used, unless otherwise stated. For ligand-dependent experiments, 1 μL of 10 μM PDS/cPDS/PhenDC3/TMPyP4 or nuclease-water was added, unless otherwise stated, after the reverse transcription buffer. The 9.5 μL mixture was heated up to 50 °C, and 0.5 μL of Superscript III or AMV was added to make up the 10 μl reaction. The reverse transcription was maintained at 50 °C for 15 min, and then 0.5 ul of 2M NaOH was added at the end of the step. The temperature was immediately ramped up to 95 °C for 10 min to inactivate the SSIII/AMV and degrade the RNA template.

For RTS, 10 µL of 2X stopping dye solution which contains 20 mM Tris, pH 7.5, 20 mM EDTA, 94% deionized formamide was added to the reaction mixture. Orange G dye was added as tracker. For RTS-HBLMPCR, 1 µL of 1 M Tris pH 7.5 and 10 µL of nuclease-water was added, followed by spin column purification by Biorad P6 column. The sample was then subjected to single-stranded DNA (ssDNA) ligation.

Single-stranded DNA ligation
To set up the ssDNA ligation, 18 µL of cDNA sample was added to 1 µL of 100 µM ssDNA linker. The mixture was heated at 95 ^{o}C for 3 min, and cooled down at room temperature. 20 µL of 2X quick ligation buffer was added, followed by 1 µL of quick T4 DNA ligase (NEB). The reaction mixture was mix thoroughly and incubated at room temperature for 2 h. Reaction was heat inactivated at 80 ^{o}C for 15 min and DNA was extracted by phenol-chloroform followed by microspin S200 column purification.

Gene-specific PCR
To set up the PCR, 1 µL of 10 µM linker-specific forward primer and 1 µL of 10 µM Cy5 fluorescently-labelled gene-specific primer was added to 10.5 µL of ligated cDNA, and 12.5 µL of 2X KAPA HiFi HotStart ReadyMix was added to make up a 25 µL PCR reaction. The PCR cycle is as follow: 95 ^{o}C : 3 min, (98 ^{o}C : 25 s ; 62 ^{o}C : 15 s ; 72 ^{o}C : 30 s)$_{25}$, 72 ^{o}C : 5 min. Equal volume of 2X stopping dye solution which contains 20 mM Tris, pH 7.5, 20 mM EDTA, 94 % deionized formamide was added to the reaction mixture. Orange G dye was added as tracker.

Data collection and analysis
RTS and RTS-HBLMPCR samples were subjected to size fractionation by 8.3 M urea 8 % polyacrylamide sequencing gel. The power was kept at 95-100W for 1-1.5 h. The surface temperature was about 50-60 ^{o}C to ensure denaturation of DNA. The gel was scanned with phosphorimager, using fluorescence setting for Cy5, with 633 nm red laser and 670 nm BP30 filter, with 3 mm platen. The gel was analysed by ImageQuant 5.2 and data was processed by excel.

ssDNA ligation efficiency test
To assess the ligation efficiency, the same procedures were followed as described above in reverse transcription and ssDNA ligation section. 1 µg of total cellular RNA and 2.5 µM of 5.8S rRNA reverse primer were used. A no T4 DNA ligase control was performed in parallel.

Quantitative reverse transcription PCR (qRT-PCR)
The qRT-PCR was performed as per manufacturer protocols. Total cDNA was prepared from 100 ng of total cellular RNA with the Superscirpt III first-strand synthesis system for RT-PCR (Life Technologies, Invitrogen). Random hexamer was used for the reverse transcription. The qRT-PCR was performed by using iQ™ SYBR® Green Supermix (Bio-Rad), cDNA template, and gene-specific primer sets designed by IDT primer design tool. No reverse transcriptase and no template controls were performed in parallel to check for DNA contamination and primer-dimer. Primers for *5.8S* rRNA (NR_003285.2), *ACTB* mRNA (NM_001101.3), and *TERC* lncRNA (NR_001566.1) are provided in Table S1. Threshold cycle (Ct) values in qRT-PCR experiments were averaged across two biological replicates. The averaged Ct value was used for the calculation of relative transcript expression.

Table S1. DNA and RNA sequences used in this study.

Name	Sequence (5'-3')
NRAS DNA template	GAACCGCACCGAAGCGCGATTTGCCCAGACCCGCCCCTCCCTTGGTCCGAAGACCTATAGTGAGTCGTATTA
TRF2 DNA template	GAACCGCACCGAAGCGCGATTTGGCCCTCCCCGCCCTCCCGTTGGTCCGAAGACCTATAGTGAGTCGTATTA
MT3 DNA template	GAACCGCACCGAAGCGCGATTTGTCCCTCTCCCTCCCTCCCTCTTGGTCCGAAGACCTATAGTGAGTCGTATTA
BCl2 DNA template	GAACCGCACCGAAGCGCGATTTGCCCCAGCTCCCACCCCACGGCCCCCTTGGTCCGAAGACCTATAGTGAGTCGTATTA
ADAM10 DNA template	GAACCGCACCGAAGCGCGATTTGCCCCTACCTCCCGCCCCTACCCGTCCCCCTTGGTCCGAAGACCTATAGTGAGTCGTATTA
ZIC1 DNA template	GAACCGCACCGAAGCGCGATTTGCCCCGGCCTCCCCCGCCCCCCCCACCCTTGGTCCGAAGACCTATAGTGAGTCGTATTA
TERRA DNA template	GAACCGCACCGAAGCGCGATTTGTAACCCTAACCCTAACCCTAACCCTAATTGGTCCGAAGACCTATAGTGAGTCGTATTA
TERC DNA template	GAACCGCACCGAAGCGCGATTTGCCACCACCCCTCCCAGGCCCACCCTCCGCAACCCTTGGTCCGAAGACCTATAGTGAGTCGTATTA
Hairpin DNA template	GAACCGCACCGAAGCGCGATTTGGGTGTCTTTTACATCTATCCTTTGATGCACACATTGGTCCGAAGACCTATAGTGAGTCGTATTA
Pseudoknot DNA template	GAACCGCACCGAAGCGCGATTTGCTGTAGGCTCGCTTTGCAAGGGGTACCAGCCGAGCCTGACTGATACCCCAAGCTTCTTGGTCCGAAGACCTATAGTGAGTCGTATTA
TAR hairpin DNA template	GAACCGCACCGAAGCGCGATTTGGGTTCCCTAGTTAGCCAGAGAGCTCCCAGGCTCAGATCTGGTCTAACCAGAGAGACCTTGGTCCGAAGACCTATAGTGAGTCGTATTA
T7 promoter	TAATACGACTCACTATAG
RP for IVT RNA	/5Cy5/ GAACCGCACCGAAGCGCG
RP for *5.8S* rRNA	/5Cy5/ AAGCGACGCTCAGACAGG
Outer RP for *TERC* lncRNA	GCTCTAGAATGAACGGTGGAAG
Nested RP for *TERC* lncRNA	/5Cy5/ TACGCCCTTCTCAGTTAGGGTTAGAC
ssDNA linker	/5Phos/ AGATCGGAAGAGCGTCGTGTAGCTCTTCCGATCTNNNNNN /3SpC3/
FP for RTS-HBLMPCR	CTACACGACGCTCTTCCGATCT
qFP for *5.8S* rRNA	GCTAGCTGCGAGAATTAATGTG
qRP for *5.8S* rRNA	GCTCAGACAGGCGTAGC
qFP for *ACTB* mRNA	GGATCAGCAAGCAGGAGTATG
qRP for *ACTB* mRNA	AGAAAGGGTGTAACGCAACTAA
qFP for *TERC* lncRNA	TTTGTCTAACCCTAACTGAGAAGG
qRP for *TERC* lncRNA	CTCTAGAATGAACGGTGGAAGG
DNA G4 (*c-MYC*)	TTGA**GGG**T**GGG**TA**GGG**T**GGG**TAA
NRAS IVT RNA	GGUCUUCGGACCAA**GGG**A**GGGG**C**GGG**UCU**GGG**CAAAUCGCGCUUCGGUGCGGUUC
TRF2 IVT RNA	GGUCUUCGGACCAAC**GGG**A**GGG**C**GGGG**A**GGG**CCAAAUCGCGCUUCGGUGCGGUUC
MT3 IVT RNA	GGUCUUCGGACCAAGA**GGG**A**GGG**A**GGG**AGA**GGG**ACAAAUCGCGCUUCGGUGCGGUUC
BCl2 IVT RNA	GGUCUUCGGACCAA**GGGGG**CCGU**GGGG**U**GGG**AGCU**GGGG**CAAAUCGCGCUUCGGUGCGGUUC
ADAM10 IVT RNA	GGUCUUCGGACCAA**GGGGG**AC**GGG**UA**GGGG**C**GGG**A**GG**UA**GGGG**CAAAUCGCGCUUCGGUGCGGUUC
ZIC1 IVT RNA	GGUCUUCGGACCAA**GGG**U**GGGGGGGG**C**GGGGG**A**GG**CC**GGGG**CAAAUCGCGCUUCGGUGCGGUUC
TERRA IVT RNA	GGUCUUCGGACCAAUUA**GGG**UUA**GGG**UUA**GGG**UUA**GGG**UUACAAAUCGCGCUUCGGUGCGGUUC
TERC IVT RNA	GGUCUUCGGACCAA**GGG**UU**G**C**GG**A**GGG**U**GGG**CCU**GGG**A**GGGG**U**GG**U**GG**CAAAUCGCGCUUCGGUGCGGUUC
Hairpin IVT RNA	GGUCUUCGGACCAAUGUGUGCAUCAAAGGAUAGAUGUAAAAGACACCCAAAUCGCGCUUCGGUGCGGUUC
Pseudoknot IVT RNA	GGUCUUCGGACCAAGAAGCUUGGGGUAUCAGUCAGGCUCGGCUGGUACCCCUUGCAAAGCGAGCCUACAGCAAAUCGCGCUUCGGUGCGGUUC
TAR hairpin IVT RNA	GGUCUUCGGACCAAGGUCUCUCUGGUUAGACCAGAUCUGAGCCUGGGAGCUCUCUGGCUAACUAGGGAACCCAAAUCGCGCUUCGGUGCGGUUC

Footnote: Green, red, and blue text indicates T7 promoter sequences, 5' hairpin sequences, and 3' hairpin sequence respectively. Underlined text is the region of interest. The Gs that can be involved in G-quadruplex formation are in bold.

Table S2. RTS effect of all IVT RNA constructs.

	**K^+/Li^+**	**K^+[PDS]/Li^+**	**Li^+[PDS]/Li^+**
**NRAS**	16.4 ±1.1	18.6 ±1.8	5.3 ±0.9
**TRF2**	12.4 ±0.1	11.9 ±1.5	5.4 ±0.3
**MT3**	8.6 ±0.2	8.3 ±0.1	3.1 ±0.1
**BCL2**	5.3 ±0.1	5.7 ±0.1	2.8 ±0.1
**ADAM10**	4.2 ±0.1	4.3 ±0.1	2.7 ±0.1
ZIC1* (lower)**<br>ZIC1* (upper)**	3.7 ±0.1<br>3.1 ±0.1	4.0 ±0.1<br>3.0 ±0.1	1.8 ±0.1<br>3.4 ±0.1
**TERRA**	8.7 ±0.2	10.3 ±0.1	3.1 ±0.2
TERC* (lower)**<br>TERC* (upper)**	8.3 ±0.1<br>4.1 ±0.1	7.2 ±0.1<br>6.0 ±0.6	1.7 ±0.3<br>2.5 ±0.1
**HP**	1.1 ±0.1	1.2 ±0.1	1.0 ±0.1
**PK**	1.1 ±0.1	1.2 ±0.1	1.1 ±0.1
***TAR* HP**	1.1 ±0.1	1.3 ±0.1	1.0 ±0.1

Footnote: Sequence information is listed in Table S1. Gel results can be found in Figure 4 and Figures S2-11. The RTS effect is defined as the fraction of stalling observed as a proportion of the total RT events under the condition employed (e.g. K^+, K^+[PDS], or Li^+[PDS]) over Li^+. All experiments were performed at least twice and the averaged RTS effects were reported.

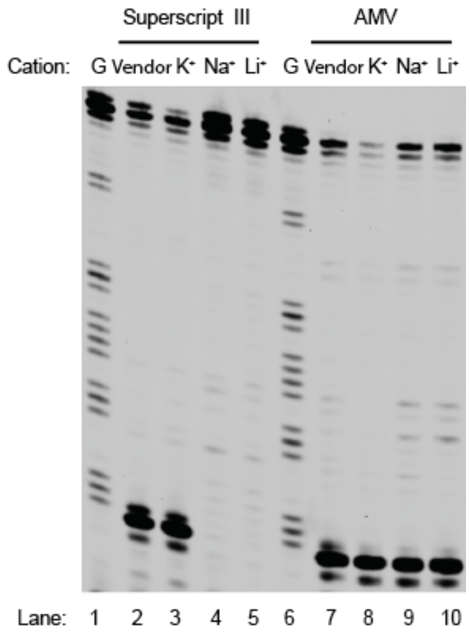

Figure S1. Identification of selective reverse transcriptase and reverse transcription conditions for RNA G4-mediated RTS. *NRAS* IVT RNA was used here. Superscript III and AMV results were shown here as examples. In lanes 1 and 6 are G ladder via dideoxyC sequencing using Superscript III. Lanes 2-5 and Lanes 7-10 show the reverse transcription results using Superscript III and AMV reverse transcriptase, respectively. A monovalent cation-dependent revere transcriptase stalling (RTS) was observed only in Superscript III, but not in AMV. The vendor reverse transcription buffer contains 75 mM K^+ final and 30 mM K^+ final in Superscript III and AMV, respectively.

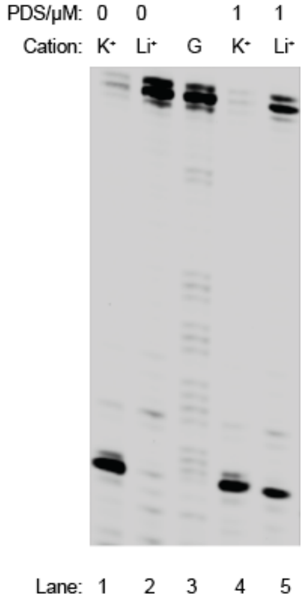

Figure S2. Cation- and PDS-dependent RTS on *TRF2* IVT RNA. Lanes 1 and 2 show the reverse transcription results under 150 mM K^+ or Li^+. Lanes 4 and 5 show the reverse transcription results under 150 mM K^+ or Li^+ with 1 μM PDS. Lane 3 is sequencing of G.

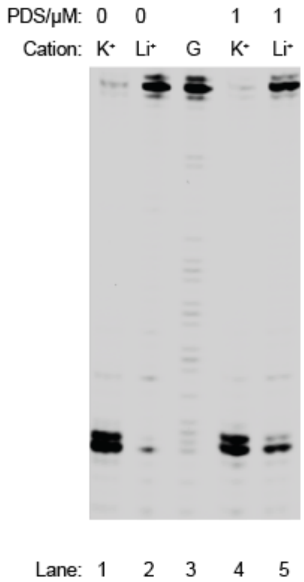

Figure S3. Cation- and PDS-dependent RTS on *MT3* IVT RNA. Lanes 1 and 2 show the reverse transcription results under 150 mM K^+ or Li^+. Lanes 4 and 5 show the reverse transcription results under 150 mM K^+ or Li^+ with 1 μM PDS. Lane 3 is sequencing of G.

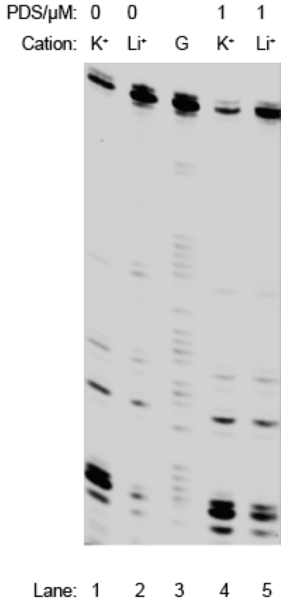

Figure S4. Cation- and PDS-dependent RTS on *BCL2* IVT RNA. Lanes 1 and 2 show the reverse transcription results under 150 mM K^+ or Li^+. Lanes 4 and 5 show the reverse transcription results under 150 mM K^+ or Li^+ with 1 μM PDS. Lane 3 is sequencing of G.

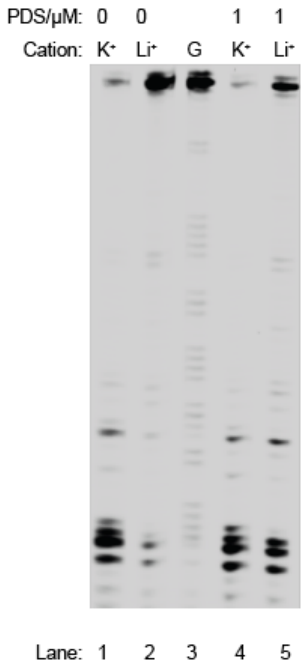

Figure S5. Cation- and PDS-dependent RTS on *ADAM10* IVT RNA. Lanes 1 and 2 show the reverse transcription results under 150 mM K^+ or Li^+. Lanes 4 and 5 show the reverse transcription results under 150 mM K^+ or Li^+ with 1 μM PDS. Lane 3 is sequencing of G.

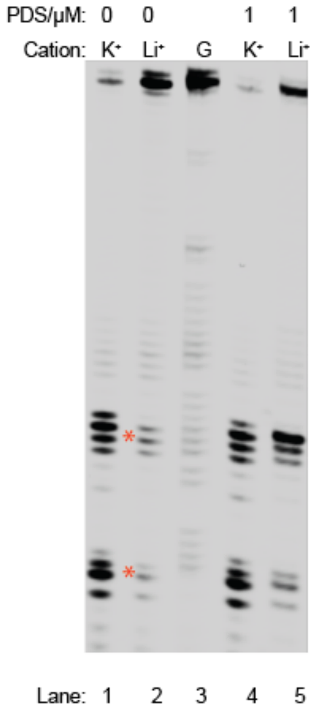

Figure S6. Cation- and PDS-dependent RTS on *ZIC1* IVT RNA. Lanes 1 and 2 show the reverse transcription results under 150 mM K^+ or Li^+. Lanes 4 and 5 show the reverse transcription results under 150 mM K^+ or Li^+ with 1 μM PDS. Lane 3 is sequencing of G. Two major stallings (lower and upper) were found (red asterisks), possibly due to the presence of more than 4 G-tracts, thus alternative G4 folds. (see Table S1).

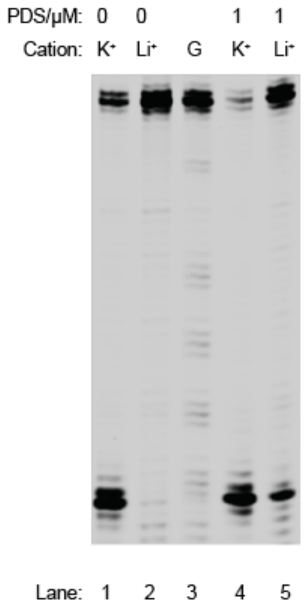

Figure S7. Cation- and PDS-dependent RTS on *TERRA* IVT RNA. Lanes 1 and 2 show the reverse transcription results under 150 mM K^+ or Li^+. Lanes 4 and 5 show the reverse transcription results under 150 mM K^+ or Li^+ with 1 μM PDS. Lane 3 is sequencing of G.

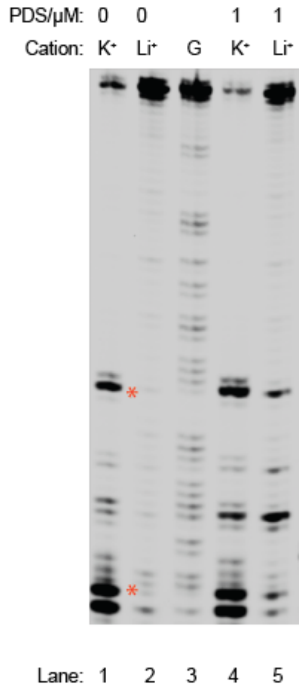

Figure S8. Cation- and PDS-dependent RTS on *TERC* IVT RNA. Lanes 1 and 2 show the reverse transcription results under 150 mM K^+ or Li^+. Lanes 4 and 5 show the reverse transcription results under 150 mM K^+ or Li^+ with 1 μM PDS. Lane 3 is sequencing of G. Two major stallings (lower and upper) were found (red asterisks), possibly due to the presence of more than 4 G-tracts, thus alternative G4 folds. (see Table S1).

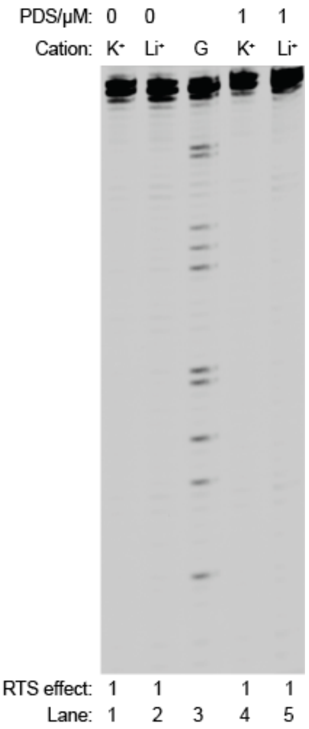

Figure S9. Cation- and PDS-dependent RTS on HP IVT RNA. Lanes 1 and 2 show the reverse transcription results under 150 mM K^+ or Li^+. Lanes 4 and 5 show the reverse transcription results under 150 mM K^+ or Li^+ with 1 μM PDS. Lane 3 is sequencing of G. The hairpin structure has been verified previously[2].

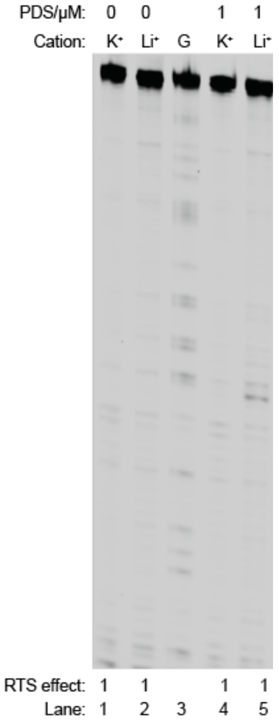

Figure S10. Cation- and PDS-dependent RTS on PK IVT RNA. Lanes 1 and 2 show the reverse transcription results under 150 mM K^+ or Li^+. Lanes 4 and 5 show the reverse transcription results under 150 mM K^+ or Li^+ with 1 μM PDS. Lane 3 is sequencing of G. The pseudoknot structure has been verified previously[3].

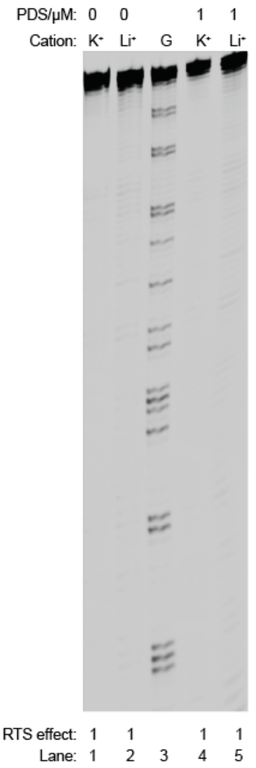

Figure S11. Cation- and PDS-dependent RTS on *TAR* HP IVT RNA. Lanes 1 and 2 show the reverse transcription results under 150 mM K^+ or Li^+. Lanes 4 and 5 show the reverse transcription results under 150 mM K^+ or Li^+ with 1 μM PDS. Lane 3 is sequencing of G. The hairpin structure has been verified previously[4].

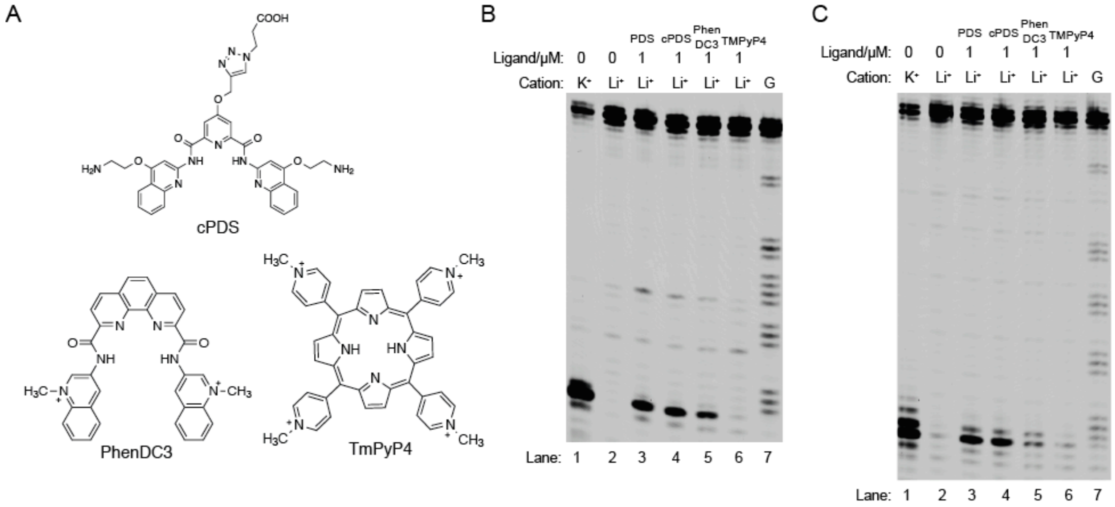

Figure S12. G4 ligand-dependent RTS on *NRAS* and *TERRA* IVT RNAs. A) Chemical structures of cPDS, PhenDC3, and TMPyP4. B-C) RTS result on NRAS and TERRA IVT RNAs. Lanes 1 and 2 show the reverse transcription results under 150 mM K^+ or Li^+. Lanes 3-6 show the reverse transcription results under 150 Li^+ with 1 μM PDS, cPDS, PhenDC3, and TMPyP4 respectively. Lane 7 is the sequencing of G.

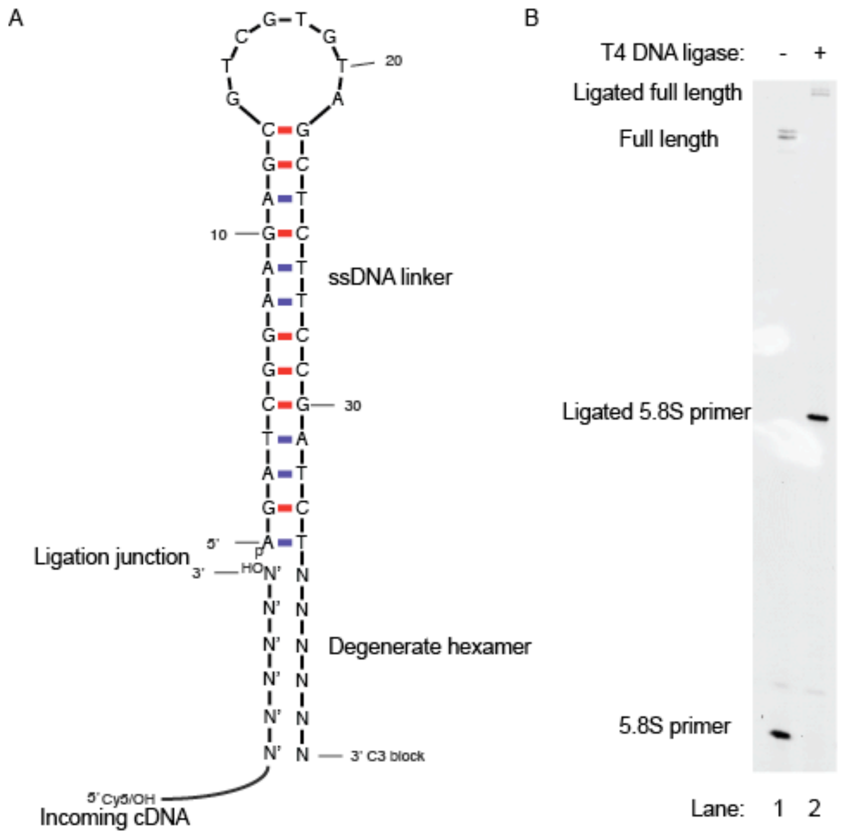

Figure S13. Single-stranded DNA (ssDNA) ligation construct and efficiency test using human *5.8S* rRNA. **A)** ssDNA ligation construct. The ssDNA linker folds into a stable hairpin as predicted by Mfold[5], and the degenerate hexamer (N6) region serve as a template to hybridize and ligate to incoming cDNA via T4 DNA ligase. **B)** Ligation efficiency test was conducted using human 5.8S rRNA. Reverse transcription was performed using a gene-specific Cy5-labeled primer, followed by ligation (see materials and methods). Lane 1 shows the control experiment in which no T4 DNA ligase was added to the reaction. Lane 2 shows the ligation results after 2 h. Both the *5.8S* primer and full length were quantitatively ligated to yield larger size products.

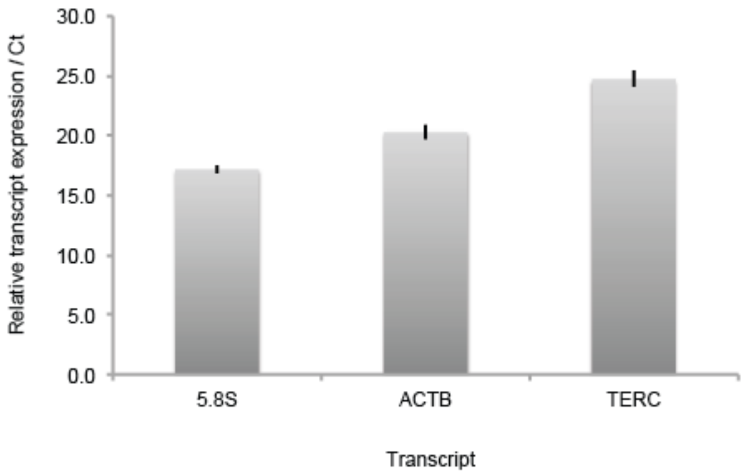

Figure S14. qRT-PCR result on human *5.8S* rRNA, actin *ACTB* mRNA and *TERC* lncRNA. The relative abundance of the three transcripts was determined by qRT-PCR. The abundance of *TERC* is much lower than *5.8S* rRNA and *ACTB* mRNA. Note that the y-axis is the threshold number (Ct) observed in qRT-PCR experiment. The relative expression between transcripts can be calculated by the following equation: $2^{(-\Delta Ct)}$.

References:

[1] C. K. Kwok, Y. Ding, S. Shahid, S. M. Assmann, P. C. Bevilacqua, *Biochem J* **2015**, *467*, 91-102.
[2] J. T. Low, K. M. Weeks, *Methods* **2010**, *52*, 150-158.
[3] M. Kozak, *Nucleic Acids Res.* **2001**, *29*, 5226-5232.
[4] J. M. Watts, K. K. Dang, R. J. Gorelick, C. W. Leonard, J. W. Bess Jr, R. Swanstrom, C. L. Burch, K. M. Weeks, *Nature* **2009**, *460*, 711-716.
[5] M. Zuker, *Nucleic Acids Res.* **2003**, *31*, 3406 - 3415.
